# Bringing facilitation into view

**DOI:** 10.1186/s40594-017-0088-x

**Published:** 2017-11-25

**Authors:** Elizabeth A. van Es, Miriam Gamoran Sherin

**Affiliations:** 1 University of California Irvine, Irvine, CA USA; 2 Northwestern University, Evanston, IL USA

## Abstract

The articles in this volume reflect the continued popularity of video in professional development and raise important questions about how to situate video productively in varied contexts. Together, they highlight the complexity of expanding video-based professional development beyond designers and the challenges that designers and facilitators experience as they prepare others to lead teacher learning in these settings. In this commentary, we examine how these papers advance research on facilitation of professional development, with a particular focus on the issues for using video productively with teachers. We conclude by raising several issues for future research.

The articles in this special issue reflect the continued popularity of video in teacher preparation and professional development and raise important questions about how to situate video productively in varied contexts. Like others, we see video as a powerful tool that has the potential to support teacher learning. Achieving this potential, however, demands attention to the system in which video is situated and to the details of how video is used (Blomberg et al., 2013; Goldsmith & Seago, 2011). These articles investigate this issue by considering the nature of facilitation within video-based professional development. In particular, they explore “What do facilitators need to know about video?” and “What do facilitators need to know about orchestrating discussions of video among teachers?” These articles also explore issues in the design of professional development materials and how to prepare facilitators to enact video-based professional development in different contexts. They ask “How might the study of facilitation inform our understanding of the key features of quality video-based materials and professional development?” and “How do materials and practices for facilitation need to be adapted for use in different settings?”

These questions are particularly important given the current context of public schooling in the USA. First of all, there have been numerous calls for improvements in teachers’ practices, and a wealth of research has documented that video-based professional development can help teachers develop new ways of seeing teaching and learning and support their efforts to enact new instructional practices (van Es & Sherin, 2008; Sherin & van Es, 2009; van Es & Sherin, 2010; Borko et al., 2008; Gaudin & Chaliès, 2015; Roth et al., 2011; Seago et al., 2014; Tekkumru-Kisa & Stein, 2015) Second, over the last decade, more teachers are being assigned to positions as school and district level instructional specialists to support teachers as they transition to the Common Core Standards (Domina et al., 2015). This shift from classroom teacher to instructional coach raises questions about the knowledge needed to support teacher learning and the central practices that are involved in doing so. At the same time, professional development providers have access to a wide array of choices regarding video—from online platforms (e.g., Edthena) and video libraries (e.g., TeachingChannel) to more and less structured video-based programs (Borko et al., 2014). Examining facilitation of video-based programs across different contexts, as these articles do, is critical in order for us to begin to understand the nature of the demands of video-based facilitation.

An emerging literature on facilitation has begun to document the knowledge and practices for leading professional development (Jackson et al., 2015; Elliott et al., 2009; Lesseig et al., 2016) with a growing body of work beginning to characterize the knowledge and practices for using video with teachers (van Es et al., 2014; Borko et al., 2014; Selmer et al., 2016; Zhang et al., 2011). What is unique about this set of articles, however, is that, as Tekkumru-Kisa and Stein explain in their preface to the special issue, the studies range in the distance from the designer’s initial work and in the degree to which they involve efforts to “scale up.” As a collection, they illustrate the complicated work of facilitation and the myriad decisions facilitators need to make during planning and enactment. At the same time, the articles attend to the system in which facilitation occurs and to the need for facilitators to attend not only to the participants in the system, but to the designers’ goals and the interests and needs of the local context. In addition, all four studies reflect professional development efforts that have been the focus of careful research and have demonstrated positive outcomes for participants, including the development of teachers’ knowledge, beliefs, and practices as well as, in some cases, advances in student learning. Studying facilitation in these contexts is therefore particularly significant given what these programs have accomplished.

## The complex work of facilitation

There is much to be learned from these articles about facilitation in video-based contexts. To start, the articles highlight the complexity of expanding professional development beyond the designers and the challenges involved for both designers and facilitators in this process. Across the articles, we identified three key components of facilitation in which challenges arose.

### Aligning one’s vision

Similar to teachers’ work with curriculum materials, facilitators need to understand in a detailed way the purpose of the professional development materials they use and what the program is trying to achieve and why (Remillard, 2005). At a broad level, this requires facilitators to understand the overarching goals of the program. In addition, they need to understand how particular tasks and tools coordinate to foster these goals. Yet, it is not so simple to align one’s own vision of a professional development program with the intended goals. The articles in this special issue add to our understanding of this challenge by illustrating several different approaches to managing this complex work. One approach is to articulate the learning goals and conceptual basis for those goals within the facilitation materials. Roth et al. (2017), for example, chose to list specific learning goals for using video with teachers within the facilitation materials. In addition, the facilitation materials describe the learning goals for students participating in inquiry-based science instruction, providing further information for facilitators concerning the program’s overall goals and objectives.

Another strategy is for facilitators to learn about a program’s goals by investigating the materials as they prepare to use them with teachers. In two of the studies, the facilitators developed a sense of the goals and vision of the programs as they studied the materials prior to implementation, either independently or in collaboration with the designers. For instance, Jacobs et al. (2017) explain that the facilitator, Hannah, took a significant amount of time to examine the materials and that doing so enabled her to learn how various aspects of the program worked together to achieve key goals. In addition, these authors describe their efforts to address Hannah’s questions and concerns in joint meetings between the facilitator and designers. Similarly, in Tekkumru-Kisa and Stein’s (2017) study, the facilitator tried to address the program’s goals as she prepared for implementation. In particular, her planning provided a context to anticipate teachers’ responses to tasks and consider how to strategically sequence the videos over the course of the program.

A third approach is to engage facilitators in the activities of the professional development program. Borko et al. (2017) explain that this approach can provide new teacher leaders with “first-hand experience of how the [PD] model works from the perspective of teacher-learners” (p. 9). Specifically, these authors developed a summer institute, in which future teacher leaders engaged in participation structures similar to those of the PSC model—solving and analyzing mathematics tasks and participating in video-based discussions similar to those they would lead with teachers. Two studies also incorporated the use of a “rehearsal” structure that is typically used in teacher education to provide pre-service teachers with opportunities to practice an instructional strategy in a modified setting (McDonald et al., 2013). Both Jacobs et al. (2017) and Borko et al. (2017) discuss rehearsals as a way to support the development of leaders’ facilitation skills, while also serving to highlight places of alignment and misalignment between a program’s goals and a facilitator’s vision for the program. The idea is that after exploring the materials as learners or participating in the scaffolded rehearsal context, teacher leaders would have a better understanding of the underlying objectives of the program.

### Selecting clips

A second component of facilitation that these articles highlight concerns the work of choosing video clips. Selecting video clips to use in professional development is a new task for most facilitators and requires specialized knowledge—knowledge of both the general affordances of video for teacher learning, as well as the specific affordances of particular videos. The articles in this special issue make an important contribution by elaborating what this work involves. Tekkumru-Kisa and Stein (2017), for example, emphasize the important role that facilitators play in both selecting and sequencing video clips prior to implementation in the TSCD-PD. They explain that the facilitator must consider how to organize video-clips “across the PD program by considering the trajectory of ideas that will be advanced” (p. 6). In particular, video clips should be purposefully sequenced in order to foster teachers’ “sensing, surfacing, and labeling of instructional factors that help teachers to maintain high-levels of student thinking” (p. 7). Thus, clip selection involves learning to identify videos that can help teachers develop each of these skills, as well as organizing the videos so that teachers build these skills over time.

For example, because the initial TSCD-PD sessions are intended to help teachers identify and “sense” instructional practices and the demands of instructional tasks, the facilitator must pick clips that can advance this goal. Midway through the sessions, the goal becomes to “surface similarities and differences in classrooms” (p. 7) according to evidence of cognitive demand. Thus, rather than simply identify the level of cognitive demand in the videos, the facilitator now aims to have participants identify contrasting features of the videos. As such, facilitators need to learn to select clips that reveal such differences. In the final sessions, the goal shifts to “labeling” key factors involved in maintaining cognitive demand, with clips being selected that elucidate instructional practices for sustaining high quality tasks. What the authors demonstrate is that using video productively is inextricably tied to the purposes of teacher learning and that the selection of clips is an integral step in this process. In addition, because video is used over the course of the program to achieve different aims, which videos are selected and how they are sequenced has consequences for the kinds of discussions that can occur and the extent to which video can help to achieve the goals of the program.

Borko et al. (2017) also address the centrality of clip selection. More specifically, as the researchers shifted the responsibility of selecting clips to teacher leaders, they saw the need to scaffold this task. They did so by narrowing the length and numbers of videos from which the clips would be selected so that leaders would be more likely to identify segments that would generate productive discussions. In addition, when supporting English Learners became a central focus of professional development, the research team had to learn new ways of looking at the videos in order to identify classroom excerpts that allowed them to see patterns of student access and participation and how particular forms of teacher questioning supported or limited students’ opportunities to learn. This required that they develop new lenses for identifying videos worthy of analysis.

The STeLLA program also provides insights into the range of considerations that are necessary when selecting a segment of video from a full lesson. Initially, facilitators use videocases that have been prepared by the development team. Over time, however, facilitators transition to using videos from participants’ classrooms. To scaffold this work, participants implement STeLLA lesson plans. In addition, the Lesson Analysis Protocol guides video analysis, and we suspect, is also useful in directing the selection of video clips. Similar to Tekkumru-Kisa and Stein’s (2017) work, because teachers view various clips in the same meeting, the facilitators must also consider how these clips work together to move the group towards the specified learning goal. Furthermore, STeLLA developers recognize that selecting clips is also tied to where teachers are in their trajectory of learning. Thus, facilitators need to consider what kinds of clips can be selected from teachers’ classrooms that will support ongoing efforts to enact STeLLA pedagogical practices, while also challenging participants to experiment with new pedagogical practices.

## Orchestrating discussion

Third, the articles highlight that orchestrating discussions requires a range of knowledge, tools, and practices to ensure that video is used productively. In the first paper in this special issue, Tekkumru-Kisa and Stein (2017) adapted the *Five Practices for Orchestrating Productive Mathematics Discussions* framework (Smith & Stein, 2011) to describe practices for leading video-based professional development. The adapted framework identifies the preparatory work required for facilitating video-based professional development, including setting goals and selecting tasks, anticipating teacher responses to videos, and strategically sequencing video clips for analysis, as well as in-the-moment practices for enacting productive discussions with video—carefully monitoring teachers’ ideas, and selecting and connecting their ideas to achieve the learning goals. In particular, the authors acknowledge that during video-based discussions “facilitators are faced with a wide variety of teacher responses to the video and must carefully listen to, interpret, and gently shape those responses toward a productive end” (p. 3). Similarly, facilitators need to press teachers’ thinking in order to cultivate critical conversations and move away from surface-level judgments that limit teachers’ opportunities to learn. This is consistent with other research that recognizes that sustaining high-quality video-based conversations requires facilitators’ keen attention to the ideas that unfold, how video is being used as a source of evidence for claims about instructional practice, and how participants work together to advance their own and each other’s learning (van Es et al., 2014). An important feature then, of the adapted five practices framework, is the dual focus on anticipating teachers’ ideas and close attention to those ideas in-the-moment of discussion. After carefully considering how teacher will respond to the selected video clips, facilitators are likely better equipped to attend to the ideas that emerge and to make choices about how to organize those ideas in the discussion in order to achieve the intended learning goals.

These concerns are no different when projects move from one site to multiple sites with many facilitators. In the case of STeLLA, Roth et al. (2017) explain that the designers developed a framework to support teachers’ discussions when they analyzed video: make claims supported with evidence from the clips, reason about those clips, and consider alternative interpretations about student thinking, learning, and instructional interactions. To ensure that teachers’ discussions follow this approach, however, requires facilitators to make many in-the-moment decisions. As Roth et al. (2017) describe, this includes decisions about what kinds of questions to ask to elicit, probe, and challenge teachers to deepen their understanding; when and how to model practices of analyzing teaching; how to make visible their own uses of STeLLA strategies in the professional development meetings—all while attending to whom is participating and how teachers are working together, ensuring that all participants have a voice, and monitoring teachers’ developing content and pedagogical understanding as revealed in the discussions of video. Thus, part of the preparation of facilitators includes preparing them for the in-the-moment decision-making to lead productive discussions with video so that the affordances of video are leveraged to advance teacher learning.

Taken together, these papers further elaborate the range of knowledge, skills, and practices that comprise the work of facilitation with video. At the same time, they also reveal that as programs move from the initial stage to being used more widely, the needs of facilitators change. We now turn to discuss this issue in terms of adapting materials for facilitation.

## Adaptations when moving to new contexts

An important issue that emerged in our reading of the articles concerns adapting materials for use in professional development. It is to be expected that facilitators will adapt materials based on the goals, contexts, and participants. Of interest to us, however, was that the types of adaptations seem to vary depending on a program’s position in Borko’s (2004) phases of program development and implementation. In the introduction to the special issue, Tekkumru-Kisa and Stein refer to these phases to distinguish among the different professional development programs explored in this issue. For example, in phase 1 of Borko’s model, the goal is to demonstrate that a program can promote teacher learning and to begin to document how the program design achieves that aim. At this stage then, adaptations involve researchers as designers attending carefully to particular design decisions in order to investigate the ways in which various aspects of the program foster particular learning goals. Take, for example, Tekkumru-Kisa and Stein's (2017) work with TSCD-PD, which illustrates activities taking place in phase 1. This initial work by the researchers demonstrated for them the importance of having the instructional factors associated with maintaining cognitive demand surface from the PD activities themselves instead of describing these ideas for participants.

In contrast, projects at phase 2 move from a single site to multiple sites and require a different set of considerations. In this phase, designers and facilitators work together to make sense of the design of the program and also explore how facilitators come to understand and use different features of the professional development program. For instance, Jacobs et al. (2017) collaborated with the facilitator in the preparation and enactment of the *Learning to Teach Geometry* materials, and in doing so were able to identify the kinds of adaptations that facilitators make and why they make them. The main consideration at phase 2 is about how to support new facilitators enacting the professional development with teachers. We also saw that when the programs moved to new contexts, the designers needed to attend to the broader organizational structure in which their programs were situated. For Borko et al. (2017), adaptations concerned generating tools and resources that sustained the design principles of the Problem Solving Cycle while responding to local needs. For example, to address the district’s focus on supporting English Learners, the designers created a protocol to direct teachers’ attention to issues of agency, authority, and identity when watching videos. Likewise, to address the limited experiences of the teacher leaders, they developed protocols to help leaders analyze the work of leading professional development in action.

Finally, in phase 3 of Borko’s (2004) scale-up model, projects move to multiple sites with different facilitators, which raise a host of additional issues and concerns. In the case of STeLLA, the use of the program across multiple schools and districts highlighted for designers that articulating principles and theories of learning was critical though not sufficient for facilitators to effectively use the materials. Attention to the readiness of the system in which the program would be implemented was also necessary. This work involved developing skills and supports for identifying key stakeholders in the district and establishing ongoing partnerships.

## Next steps for studying facilitation

The articles in this special issue offer particularly rich analyses that reveal the complexity of facilitation. At the same time, they raise questions for us about how to move forward with research on facilitation and how to best support future facilitators in leading professional development. Of particular interest to us is the idea of developing tools to support the work of facilitation. We think of such tools as resources that provide structure to facilitators’ planning and to their work with participants. Across the articles are important examples of protocols and frameworks that the designers developed to scaffold the observation and analysis of teaching via video. Yet, in unpacking the detailed work of facilitation, we suspect that additional tools will be needed to support facilitators, particularly in the case of facilitators new to this work. What might these tools be and what aspects of facilitation might they support? For example, we discussed the wide range of skills facilitators need to select clips and the range of considerations needed to identify a segment that will advance the specified goals. We envision that tools and frameworks that specify these considerations will be useful in coaching facilitators to select clips that feature students and teachers interacting with worthwhile content. Such a tool might identify places in a lesson where the kinds of events and interactions that lead to productive discussions take place (e.g., small group work or whole class discussions) or what should be the objects of focus in the videos for discussions to achieve different learning aims (i.e., a focus on student thinking versus a focus on participation, agency, and access).

Tools will also be needed to help leaders orchestrate discussions with video. For example, adapting the *Five Practice for Orchestrating Mathematics Productive Discussions* to facilitation implies that leaders will need tools and resources to lead discussions with video. A central tool in the original framework is a chart for monitoring student contributions (see Smith and Stein, 2011). We envision that a monitoring tool would be equally useful for facilitators leading video-based professional development. Such a tool would help them track teachers’ observations and interpretations of the videos to guide their decisions about what ideas to take up and how to work with those ideas to advance teacher learning. Alternatively, some of the tools that were developed as part of these research projects might also be useful to guide facilitators. For example, the frameworks that Jacobs et al. (2017) used to assess facilitation could be repurposed and used by a facilitator for self-reflection.

A second issue concerns facilitation of highly specified video-based programs in contrast to facilitation of more emergent and adaptive forms of video-based professional development (van Es et al., 2014). The professional development programs discussed in this special issue represent different degrees to which the program goals and resources have been defined by the designers versus in the local context (Borko et al., 2011). For example, LTG-PD studied by Jacobs et al. (2017) is highly specified, with both videos and facilitation materials determined by the designers and provided to facilitators. In contrast, STeLLA combines elements of a highly specified program with detailed information for facilitators concerning the program goals and structure, with elements of a more adaptive model in which facilitators use video from participating teachers’ own classrooms midway through the program.

While video serves as the anchor for teacher learning across these contexts, we conjecture that the facilitation demands vary. In particular, we suspect that the facilitation challenges discussed above may need to be adjusted for highly adaptive settings. For instance, in a less structured environment such as TeachingChannel or video clubs, it may be the case that designers and facilitators collaborate from the start to define the goals for using video and to design tasks that will leverage the affordances of video to achieve these goals. Additionally, when videos come from participants’ classrooms, we suspect that facilitation of conversations is different than when the videos come from others’ classrooms and that facilitators have to attend to aspects of facilitation that are relevant *because* the video is from the participants in the group and necessitates setting particular norms that may be different from viewing others’ videos (see van Es et al., 2014; Coles, 2012; Zhang et al., 2011). Because of the array of video-based environments available—with some more specified than others—we see analysis of facilitation in these different settings as a fruitful line of inquiry. Such research can lead to the identification of knowledge and practices for using video in the range of contexts, while also elucidating unique considerations for the different settings.

A final concern for future inquiry relates to the trajectory of learning for facilitators. These manuscripts reveal the work entailed in planning for and leading video-based discussions. We believe it would also be valuable to investigate the process by which facilitators come to learn to engage in these practices and how they develop knowledge for leading professional development. The question for us is, thus, not only what do facilitators need to know, but how does this learning proceed and how does the design and enactment of these programs foster this learning. Borko et al. (2017) illustrated that the researchers needed to adapt their leadership preparation program to focus on what it means to lead professional development. It was not sufficient to prepare facilitators to lead the Problem Solving Cycle; they also needed to develop dispositions and practices specific to being leaders. In the STeLLA project, the Lesson Analysis Protocol that frames video analysis removes the inclination to evaluate teaching to do careful observation and analysis to inform teaching decisions. Prior research suggests that this approach to analysis is not natural for teachers, suggesting that it is likely not natural for teacher leaders to work with artifacts in this way (van Es & Sherin, 2008; Lessieg et al., 2016). Thus, we see an important line of research that involves asking questions about what it looks like for facilitators to begin to learn to lead and how they come to shift their identities to become teacher leaders and take up practices in ways that promote teacher learning (see for example, Elliott et al., 2009). Similar to research that documents trajectories of teacher learning (see for example, van Es, 2011), findings from this work could lead to the generation of materials and resources that can be used to scaffold learning to facilitate while also lead to the development of a curriculum for facilitator learning.
